# M. Cl. Bernard on the Physiology of Absorption, and on the Abdomino-Renal Circulation

**Published:** 1854-08

**Authors:** 


"M. Cl. Bernard on the Physiology of Absorption, and on the Abdomino-Renal Circulation. Report of Lectures, with Vivisections, delivered in Paris, 1853, prepared by F. Peyre Porcher, M. D. Paris 1853—4.

We must remember that absorption diminishes in the stomach during digestion, both on account of the afflux of blood and the pouring out of the gastric juice. In inflamed tissues, also engorged with blood, absorption is much less active.

There are two varieties of absorption—the one by the chyliferous vessels, and the other by the vena porta, each of which selects peculiar substances; the election depending, in some measure, upon the necessity or not for certain of these to pass through the liver, or to proceed immediately to be eliminated by the kidneys.

Starch and sugar, we know, are absorbed into the vena porta.

Cane sugar introduced into the stomach as cane sugar, undergoes no change in the intestines into any other substance; it is absorbed as cane sugar, though a small portion of it, by acid reaction, is changed into grape sugar. In dogs and horses a very large proportion enters the vena porta in its original condition.

Milk sugar only differs from the others in submitting, with difficulty, to alcoholic fermentation; though the pancreatic juice renders it fermentable. You do not, therefore, find much of this in the vena porta.

Raisin or grape sugar is absorbed as grape sugar in the vena porta.

Cane sugar cannot serve as a nutritive substance as cane sugar; for, if you inject it into a vein, it is found eliminated as sugar, and not absorbed. But, by passing through the liver, it undergoes a transformation which renders it subservient to nutrition, by the process which converts it into grape sugar.

Now, if you inject grape sugar into the veins of the neck, you will find a part of it only in the blood, there being a certain amount of it which is really destroyed in passing. This species of sugar is also modified by the liver, which gives it a different physiological character.

Diabetic sugar, corresponding chemically with that of the grape, differs from it physiologically, as it is more easily absorbed and destroyed when injected into a vein.

The results of these observations teach us, that it is essential for all sugar to pass through the liver to undergo the fermentation and destruction, which its simple passage through the intestines is insufficient to complete.

Albumenous substances, also, M. Bernard has ascertained, must pass through the liver to receive the modifications essential to its complete absorption into the system. This is found in the lymphatics and in the vena porta, which are so difficult to experiment upon. He injected the albumen of eggs into the jugular vein, and it made its appearance in the urine; hence, of course, it does not remain as a nutritive product in the blood, though portions are also found in that fluid—(another variety of albumen, when injected in like manner, was eliminated by the kidneys)—hence both require the liver for their complete transformation. The same albumen, injected into the vena porta, passes directly to the liver, and you find none of it in the urine.*

Fatty matter does not enter the liver, as is well known, but it first passes to the lungs, when it is thrown directly into the general current of the circulation. There is a slight absorption of a small portion of it in the vena porta, but this is absorbed naturally and through no chemical change. It is not emulsionized as it is by the pancreas. This small portion, taken up by the vena porta, goes to the liver as fat, where it disappears. M. Bernard could discover no trace of its presence in the blood which had left the liver. Some physiologists sustain the opinion, that in this organ it enters into the formation of sugar; but this is not verified. It certainly disappears there, and does not pass out of the liver as fat; it may help to form bile.

So that the liver requires three kinds of substances—sugar, starch, and the small quota of fat just referred to.

*Chyliferous vessels.*—These are especially subservient to the absorption of fat. They are not found in fishes or birds: M. Bernard has fed pigeons on fat, and, upon examination, lymph was found in the vessels, but no fatty matter in suspension. In birds the latter is probably absorbed by the vena porta. In these animals the vena porta communicates largely with other veins, and there is, therefore, no absolute necessity for their contents to pass through the liver.

The liver thus appears to be interposed between the intestines and the grand circulatory system, to modify certain substances preparatory to their entrance, and prepare certain products for their elimination.

The liver has another duty to perform, viz: to prepare an equilibrium between certain substances; as, for instance, when the proportions between flesh, viands and ordinary food, varies; whether one is introduced in too great quantity, or the reverse, it is the liver which makes up for the deficiency, or converts what is superabundant into another product more congenial to the requirements of the system.

An animal, nourished exclusively on viands, has these azotized substances converted by the liver into sugar; and the exclusive diet produces no effect upon the blood proportionate to the quantity introduced by means of its reduction during its passage through this organ, which exists, as it were, as a balance to preserve from the injurious influences of excess. Its anatomical position, also, with relation to the other organs, supports this idea founded on its physiological action.

There are three maladies affecting the human economy, which depend upon different modifications of the liver and its secretion, viz: diabetes, albumenuria, and fatty or chylous urine. But these need only be referred to at present.

The whole of the ingesta is not always absorbed—a large part is carried off with the secretions which might still have served the purposes of nutrition.

The residue is composed of—1st. What is refractory to the digestive process, as corneous substances—certain epidermic coverings, insoluble phosphates, etc. 2ndly. Substances which are expelled because not digested; as when an animal is fed on fecula in excess, that only is absorbed which is converted into sugar, whilst the remainder is voided unchanged. The same is true of fatty matter in excess, which also escapes in a condition very similar to that in which it entered.

What, then, is the reaction of the coecum, that some talk so much of? The coecum, M. Bernard stated—and we saw experiments on a dog which confirmed it—has a acid reaction, but no new digestive powers. Some describe it as a second digestive stomach or cavity. The reaction of the coecum is sometimes alkaline; this depending upon the excess of certain products of digestion, or certain kinds of food introduced into the stomach. A superabundance of starch and sugar consumed by an animal, is changed to actic acid, and, when it reaches the coecum, of course presents an acid reaction. Exactly the reverse occurs if the animal had been fed on azotized substances. The coeca of carnivorous animals, consequently, present an alkaline reaction which can be reversed by altering the character of the diet.

The coecum is not, then, an organ of secondary digestion. The well known phenomena of spontaneous decomposition which occurs there, depend upon extrinsic causes. M. Bernard does not account for the odour which characterizes this portion of the intestinal tube. The great variety in the size, and the absence even of the coecum in some animals, is another strong argument to prove its comparative unimportance. In the horse it is very large, and in the dog quite small.

All substances absorbed, which pass through the liver, do not go directly to the general circulation. They seem to be arrested by this organ.

Through the kidneys, also, many substances enter without making the circuit of the general circulation. There appears to be a peculiar abdomino-renal circulation, by which the quality of urine is altered independently of any influences derived from the circulation through the heart. In many animals a great deal of fluid substances go directly to the renal vein without reaching the liver. In almost all animals, with the exception of the mammifferous, there is a considerable amount of substances ingested, which circulate through the kidneys without even coming in contact with the lung.

In some animals the kidney is really an organ with functions, varying considerably from that of others. A carnivorous animal, during digestion, has the character of its urine materially altered in the proportion of its constituent. There being two species of normal urine—that secreted by the veins, and that eliminated from the arteries—they must, of course, vary with the ingesta.

M. Bernard, to test some of the changes which food of various kinds undergoes during digestion, and to obtain some of the chyle of the thoracic canal, placed a dog upon the table which had been fed on mixed food, composed of starch, sugar, viands, fat, etc. It was then killed by passing a sharp pointed bistoury into the medulla oblongata, and, upon examination, the intestinal canal presented the following peculiarities: The chyle of the thoracic canal had a whitish appearance, like emulsionized fat. The stomach gave an acid reaction, and he found starch in it. This reaction was traced some distance in descending the intestinal tube. Little green particles of albumen, precipitated by bile, could be seen in the duodenum, which gradually disappeared lower down. The coecum was veritably alkaline.

There is a relation between the circulation through the kidneys and vena porta (abdomino-renal circulation) and the general circulation, which varies under certain circumstances—as during the absence and presence of food in the stomach—and which it is important to observe.

An animal which has, for a long time, been abstinent, receives a large quantity of food in its stomach; a draught is suddenly made upon the vena porta, which differs from other veins in its not only carrying all the blood of the mesenteraic veins, but it is also, at the same time, charged with the carrying off almost all the substances absorbed from the intestines; in this respect resembling the pulmonary artery.

Now in some animals, as in horses for example, there are veins passing directly from the vena porta to the vena cava inferior; and, when the former is much swollen, a portion of its contents pass to the latter without traversing the liver. So that just below the diaphragm is often very much distended, on account of the accumulation. A very small portion of its contents pass to the heart; the valves existing there (preparations of which were exhibited) prevent it from descending, and it is forced to reflow through the renal veins to the kidneys. So that, indeed, the kidney is between two pressures; and the secretion of urine must depend somewhat upon whether the animal is or is not in full digestion. The acid of the urine becomes alkaline during digestion, and its secretion is much increased in quantity.

During this pressure upon the valves of the vena cava, of which we have just spoken, the blood of the lower limbs goes by the vena azygios. The latter in these animals (the horse, for example) is very large, to compensate for the varying demands made upon it. After the hurry of digestion is over, the vessels cease in a measure to expand, and the blood then reflows along the vena cava, and finally gains the heart as ordinarily. But we see from the above, that a certain quantity of alimentary substances goes directly to the kidney, without reaching the heart. And the same is true of man, in whom, though with shorter intestines, it is less distinctly observed. M. Bernard was enabled to study the process with more advantage in animals with a larger and longer intestinal tube. By this view we see in what way the kidneys are enabled to maintain a balance in the circulation.

If prussiate of potash is given to an animal during full digestion, it is absorbed and passes through the hepatic and renal circulation, by which it is eliminated; a very small portion, if any, reaching the heart. A reverse condition takes place if another, which has fasted for some time, is fed on the same substance; the greater portion of it is received into the torrent of the circulation, by means of which it is conveyed to distant parts of the system. This explains the variety in the action of the same or a smaller dose of a poison, when received into the system at different times; in the one case quite a large quantity may be eliminated with the urine, and rendered innocuous, when a much smaller amount, swallowed on an empty stomach, would have been attended with dangerous or fatal consequences.

Now, the physiological fact, that at certain digestive periods a larger portion of the absorbed aliment passes to the kidney without reaching the heart, receives anatomical development in some animals in whom the vessels, which contribute to this, are in more constant service, and become proportionally enlarged. The renal veins have a very important role to play, and one can witness, by making a small aperture into the abdomen, the reflux through them. In the English race-horse they are very much increased in size, from the constant rapidity with which the circulation is often kept up. The same excessive development is observed at the *abattoirs*, among animals which have been worried or run down; in which case there is considerable hepatic engorgement, with increase in the demands made upon the portal veins, together with those contributing to the functions of the kidney. [We know, also, that if the vena porta is injected after death, it produces considerable expansion on the liver; being invested by the capsule of glisson, its walls are not adherent to the liver like the hepatic vein, and thus admit of an increase in size.]"—*Charleston Med. Jour.*

